# 

**Published:** 1859-09

**Authors:** 


EDITED BY

**JOSEPH P. LOGAN, M. D.,**

Professor of Physiology and Diseases of Women and Children in the Atlanta Medical College.

AND

**W. F. WESTMORELAND, M. D.,**

Professor of the Principles and Practice of Surgery in the Atlanta Medical College.

(Sept. 1859 — Aug 1860.)

41040

PAX ET SCIENTIA, SED VERITAS SINE TIMORE.

LIBRARY

ATLANTA, GA:

ATLANTA INTELLIGENCER PRINT,

1860.

### INDEX.

PAGE.

A

A Case of Hernia-Cerebri or Congenital Encephalocele, .... 46
Atlanta Medical College—close of the session, .... 60
An Inquiry into the Character of Green and Malænial Discharges from the Bowels, .... 92
Atlanta Medical College Commencement, .. 113
Atlanta Mineral Spring, .... 116
Alleged Abortion—Inquest by the Coroner, 124
Acetic Acid in Erysipelas; by Wm. Hauser, M. D., Spier's Turn-Out, Ga., .... 142
A Visit to Philadelphia and New York, ... 181
A Case of Morbid Growth of the Gums; by J. P. H. Brown, Dentist, Atlanta, Ga., .. 212
Abdominal Tumors Mistaken for Ovarian, 237
A Visit to Philadelphia and New York, ... 241
A New Theory of Atmospheric Temperature, .... 407
A Medico-Legal Review of the Case of Patrick Maude, .... 428
Atlanta Medical College, .... 447
A Medico-Legal Review of the case of Patrick Maude, .... 481
Address of Francis S. Colley, M. D., late President of the Medical Association of Georgia, upon retiring from the Chair, .. 527
A Glance at Medical Science among some of the East Indian Nations. From the French of Dr. L. S. Heymann, .... 538
Animal Chemistry and its Relation to Therapeutics; by J. L. Teed, M. D., of Mendota, Ill., .... 548
Arsenical Poisoning by Paper-Hangings, .. 560
American Medical Association, .... 561
Arsenic in Mennorrhagia, Leucorrhœa, &c. By Arthur Burns, M. D., of Elliot's Mills, Maryland, .... 572
Adulteration of Food and Drugs, .... 575
Address, Introductory to the Sixth Course of Lectures in the Atlanta Medical College; by J. G. Westmoreland, M. D., Professor of Materia Medica, Therapeutics, and Medical Jurisprudence, .... 577
Atlanta Medical College, .... 624
A Temperate Life, .... 627
An Essay read before the Medical Association of the State of Georgia, at their Annual Meeting, at Rome, Ga., April 11th, 1860. By Robert Southgate, A. M., M. D. "*Nil remedium, nisi tempestivo usu sit.*" .... 641
American Medical Association. Thirteenth Annual Meeting, held at New Haven, June 5th, 6th and 7th, 1860, .... 688
American Medical Association and Convention of Medical Teachers, .... 701
American Medical Association. Thirteenth Annual Meeting, held at New Haven, June 5th, 6th and 7th, 1860, .... 727
American Medical Association, .... 752

B

Battey, Robert, M. D., .... 61
Bile—Its Offices in Health, Abnormal Effects, &c.; a Thesis presented to the Faculty of the Atlanta Medical College. By J. E. Blackshear, Marietta, Ga., .... 75

PAGE.

Births, Deaths and Marriages in England, 191
Books, &c., Received, .... 247
Bibliographical, .... 366
do .... 502

C

Case of Hydrophobia, .... 21
Case of Needle-swallowing, .... 126
Case of Rupture of the Uterus with Operation. By R. A. T. Ridley, A. M., M. D., of LaGrange, Ga., .... 193
Case of Abortion, .... 229
Comparative Advantages of Ether and Chloroform, .... 250
Consumption. By J. G. Westmoreland, M. D., Atlanta, Ga., .... 273
Case of Puerperal Convulsions, .... 289
Case of Hydrophobia successfully treated with drachm doses of Calomel, .... 292
Consumption. By J. G. Westmoreland, M. D., Atlanta, Ga., .... 404
Criminal Abortion, .... 442
Closing Address of Dr. Geo. B. Wood, to the Class of the Medical Department of the University of Penn., Feb. 29th, 1860, 562
Cultivation of "Ginseng," .... 565
Cholera Infantum. By A. W. Griggs, M. D., of West Point, Ga., .... 589
Convention of Medical Teachers, .... 753
Close of the Volume, .... 754

D

Dr. Corson's New Physical Signs, .... 63
Demoralization a Source of Disease, .... 110
Dr. Hillyer's Address, .... 115
Discovery of the Anæsthetic Effects of Chloroform, .... 119
Detection of Pregnancy by Ergot, .... 127
Dissection Wounds, .... 493
Death of a Medical Practitioner by Chloroform, .... 625
Death from Inhalation of Chloroform, in Lisbon, .... 636
Dr. Hay's Arctic Expedition.—The Views of its Commander, .... 756
Doctors and Physic, .... 759

E

Easy method of Extracting Foreign Bodies from the Eye-lids, .... 123
Exodus of Southern Medical Students, .... 248
Editorial Correspondence of the Peninsular and Independent Medical Journal, .... 326
Editorial Correspondence, .... 363
Ether as an Anæsthetic agent, .... 379
External Manipulations of Fœtus in Utero, to rectify supposed malpositions, &c., .. 416
Editorial Correspondence, .... 434
Employment of Iodide of Potassium in the Treatment of Aneurisms, .... 638
Extraction of a Fragment of a Silver Catheter from the Bladder, by the Operation of Lithotomy, .... 639

F

Florence Nightingale and her "Notes on Nursing," .... 509
Franking of Vaccine Virus, .... 627
Fracture of the Skull in Natural Parturition, .... 638

### INDEX TO THE FIFTH VOLUME MEDICAL & SURGICAL JOURNAL.

PAGE.

G

Gun-Shot Wound. By J. S. Weatherly, M. D., of Montgomery, Ala., .............. 139
Georgia Medical and Surgical Encyclopedia, .............................. 376

H

Hypophosphites in Tuberculosis, .. ..... 62
Hygienic and Literary Magazine, ......... 188
Hygiene in the Practice of Medicine. By V. H. Taliaferro, M. D., Atlanta, Ga., .. 207

I

Intra-Uterine Fractures, .......... . .... 536
Items, .................................. 640
Index to Volume Fifth, ..... ........ .... 767

L

Ligature of the Common Iliac Artery for Aneurism—Use of Silver Ligature, ....... 102
Labor, with the Hymen unbroken, ........ 111
List of Payments, ..... ....... ......... 128
Letters from Professors J. Hughes Bennet, of Edinburgh, and Carl Rokitansky, of Vienna, on the Post-Mortem Examination of the late S. S. Whitney, ........... 143
Long Island College Hospital, Brooklyn, N. Y., ........ ...................... 377
Lobelia—Ism—Its prospects and policy, .. 381
Liebig's Soup for Invalids, ........ ...... 626

M

Martyrs of the age, ................. .... 26
Monthly Review of Parisian Medicine and Surgery, ........................... 30
Meteorological Table, ................... 64
Medical Department of the University of the Pacific, .......................... 115
Meteorological Table, ................... 128
Memoir on the Hybridity of Animals, . ... 166
Modern Mental Disorders in a Medico-Legal Point of View, ..... ............ 185
Meteorological Observations, ............ 192
Medicinal Garden, ................. .. .. 252
Means of Preventing the Pitting of Small Pox, ................................ 255
Meteorological Table, ....... ........... 256
Medical Reminisnences of New York. By D. C. O'Keefe, M. D., Atlanta, Ga., .... 279
Medical Association of Georgia, .... ..... 377
Medical College of South Carolina, ....... 377
Meteorological Table, January, .......... 383
do do November, ..... ... 384
Medical Reminiscences of New York. By D. C. O'Keefe, M. D., Atlanta, Ga., .... 394
Medical Association of Georgia, .......... 447
Meteorological Table, .... ........... 448
Medical Reminiscences of New York. By D. C. O'Keefe, M. D., Atlanta, Ga., .... 465
Mortality of Sacramento City, Cal., for 1859, ........... ..... ............... 507
Meteorological Table, ................... 512
Mutilation of a Child by a Homœopathic Physician. By Hiram Nance, M. D., La-Fayette, Ill., ........................... 546
Medical Association of Georgia, .......... 561
Meteorological Table, ... ................ 576
Metropolitan and Central Medical Ethics, . 702
Medical Journalism, ........ ..... .. ... 703
Meteorological Observations, ............. 704
Medical Education, ...................... 755
Meteorological Observations, ............ 768

PAGE.

N

Notice of Memorials concerning the treatment of Pulmonary Phthisis, ........... 41
New York Correspondence, ............... 49
New York Correspondence, ............... 62
New Mode of Relieving Retention of Urine 124
New York *vs.* Philadelphia, ......... .. 191
New Medical Journal, ............ ...... 254
Notes of a Visit to Lunatic Asylums in Great Britain and Ireland, .............. 304
Natural History Specimens, ............... 640
New Orleans School of Medicine, ......... 754

O

Odontalgia. By J. P. H. Brown, Dentist, Atlanta, Ga., ....................... 71
Our Journal, ........................... 49
On the state of the Nutritive Functions during the progress of Continued Fever, .. 158
On the Use of Potash in some Cutaneous Diseases, ..................... .... 175
Our Journal, ............................ 247
On the Production of Cataract in Frogs, by the administration of Sugar, ........... 320
Our Paris Correspondence, ............... 385
On the Local Employment of Chloroform in the Reduction of Dislocations, ... ...... 574
On the Causes of the Death of Animals subjected to High Temperatures, .......... 628

P

Professional Ollapodrida. By a Retired Pharmacien, .......................... 1
Physiology of Menstruation. By Thomas S. Denny, M. D., Atlanta, Georgia, ..... 18
Prevention and Treatment of Mental Disorders, ........ .................... 148
Preparatory Course, ................... ... 190
Phrenology. By Jos. W. Clift, of Atlanta, Ga., ................. ............ .. 270
Payments, .... ............................ 382
Phrenology. By V. H. Taliaferro, M. D., of Atlanta, Ga., ......... ............. 449
Prevention of the Unpleasant Taste of Balsam Copaiva, ... .. ... .............. 569
Pyrin, ............... ................ 570
Prof. W. F. Westmoreland's New Instrument, ....... ........................ 572
Preservative Fluid, ...................... 575
Preservation of Bodies for Anatómical purposes, .... ................. .... 628
Phrenology. By J. W. Clift, of Columbus, Ga., ............................. 683

Q

Quackery and its Cure. By A. G. Thomas, A. M., M. D., of Atlanta, Georgia, ....... 82
Quinio, .... ......... .. ............. . 571
Quackery Rampant, ...................... 767

R

Remarkable disappearance of a Pessary, and its subsequent Removal from the Rectum, ............................... 35
Raw meat in Diarrhœa of Children, ........ 63
Replacement of an Extracted Tooth, ..... 112
Remarkable Luxation of the Eye, ......... 126
Remarks on Vineyards. By an "Observer," ................................ 274
Review of the Emotions and the Will, ..... 343
Reports of Cases occurring in "Bellevue Hospital," Richmond, Va. By Thomas Pollard, M. D., one of the Physicians, .. 458
Report of the Proceedings of the Medical Association of Georgia, for 1860, ........ 513

PAGE.

Report of a Case of Rupture of the Uterus, presented to the Medical Association of Georgia. By S. W. Burney, M. D., Forsyth, Ga.,. .......................... 521
Report of a Case of Ulceration of the Cervix Uteri, read before the Medical Association of Georgia. By A. M. Boyd, M. D., of Cave Spring, Ga.,................ 531

S

Successful repetition of Wurtzer's method for the Radical Cure of Inguinial Hernia in a case of 20 years' standing. By W. C. Smith, M. D., of Grantville, Ga.,..... 17
Scarlatina after Parturition,.. ......... 127
Southern Students in Northern Medical Colleges,................ .. .. ..... 253
Subterranean Fire in Belgium,........... 256
Scarlet Fever, . ......... ... ....... .. 378
Southern Medicine, ........... ......... 445
Santonate of the Protoxide of Mercury—an excellent Vermifuge,............ .. 566
Syria as a Residence for Consumptives,... 579
Singular Effects of Snake Poison,......... 629
Summer Schools,......................... 630
Severe Burn of the Neck, treated by extension from the beginning,............ 637
Sabbath Clinical Benevolence. By Alex. Means, M. D., Oxford, Ga.,. .......... 716

T

Two Obstetrical Cases in Dwarfs. By A. J. Shaffer, M. D., of Lawrenceville, Ga., 65
Typhoid Fever,......................... 117
To Subscribers, Correspondents, &c.,..... 190
The Physician's Hand-Book of Practice for 1860,................................. 190
The Life and Labors of Lænnec: An Introductory Address delivered at the New Orleans School of Medicine, November, 14, 1859,............ ...... . . 214
The Mouth to Mouth *vs.* The Marshall Hall Method in Asphyxia Neonatorum,...... 232
Transactions of the American Medical Association. Vol. XII, 1859,.............. 246
To Subscribers,................. ......... 247
The Age of Man,.... ............ ....... 251

PAGE.

The New Medical School at Chicago,...... 254
To Subscribers,........ ................. 376
The Present Issue,................ .. .. 376
The Cholera in Germany,......... . ..... 447
Typhoid Fever—Nathan Smith, &c.,...... 566
The Law of Rape—Chloroform in Rape Cases. Reported by M. B. Walker, Esq., Attorney for Defendant, with remarks by J. C. Reeve, M. D., both of Dayton, Ohio,.................................. 607
Tasteless Medicines. By J. W. Thompson, M. D., Philadelphia,... .............. .. 621
The Epidemic among Cattle,. ........... 627
The Influence of the Placenta on the Development of the Uterus during Pregnancy,................................. 633
Tetanus, ........ .................. ..... 635
Typhoid Fever. By V. H. Taliaferro, M. D., Columbus, Georgia,................ 705
The Vaccination of Indians,............... 759

U

University of the Pacific—Medical Department,........................... ...... 559
Unguentum Glycerini,..................... 570

V

Valuable Surgical Discovery,............. 121
Valedictory Address to the Graduates of the Atlanta Medical College for the year 1859. By Eben Hillyer, M. D., of Rome, Georgia,............................. 129
Valedictory Address upon the part of the Class, 1859, in the Atlanta Medical College. By B. F. Ward, of Miss.,........ 257
Vaccination, ............................. 567
Vital Forces—The Closing Lecture of the Course on Chemistry, to the Students of the Savannah Medical College, delivered March 10th, 1860. By N. A. Pratt, M. D., 596

W

Who discovered Anæsthesia?............ 107
Winter Course of Lectures,............... 114
Why are we not a Healthy People? By Chas. H. Bass, M. D., Assistant Physician to the Georgia State Lunatic Asylum, Milledgeville, Ga.,................. 197

